# Procalcitonin and D-dimer for Predicting 28-Day-Mortality Rate and Sepsis Severity based on SOFA Score; A Cross-sectional Study

**DOI:** 10.29252/beat-070404

**Published:** 2019-10

**Authors:** Zeinab Naderpour, Mehdi Momeni, Elnaz Vahidi, Javad Safavi, Morteza Saeedi

**Affiliations:** 1 *Department of Internal Medicine, Shariati Hospital, Tehran University of Medical Sciences, Tehran, Iran*; 2 *Prehosptal and Hospital Research Center, Tehran University of Medical Sciences, Tehran, Iran*

**Keywords:** Fibrin fragment D, Mortality, Procalcitonin, Prognosis, Sepsis

## Abstract

**Objective:**

To determine the possible relationship of procalcitonin (PCT) and D-dimer with the 28-day-mortality rate and severity of sepsis based on sequential organ failure assessment (SOFA) score.

**Methods:**

In this cross-sectional study, patients were enrolled based on their signs and symptoms of sepsis confirmed by essential laboratory studies. Demographic data, Glasgow coma scale and vital signs, serum PCT and D-dimer levels, creatinine, bilirubin level, arterial blood gas analysis and platelet count were recorded. Disease severity index was assessed based on SOFA score. Patients’ 28-day-mortality rate and hospital length of stay were compared with the study variables.

**Results:**

Sixty-four patients with the mean age of 78.3±11.6 were included of whom 34 cases (53.1%) were male. The 28-day-mortality rate was 17%. The analysis showed that only patients’ age (*p*=0.01) and platelet count (*p*=0.02) had a statistically significant association with the mortality rate. SOFA score had no statistically significant correlation with PCT or D-dimer; and these two markers didn’t have any significant correlation in terms of predicting mortality due to the sepsis.

**Conclusion:**

In our study, PCT and D-dimer failed to show any significant correlation with 28-day-mortality rate of sepsis.

## Introduction

Sepsis is a growing problem that has a high mortality rate (60%). The disease burden is a true catastrophe especially in children, elderlies and immunocompromised patients [1-4]. Most studies have defined sepsis as a disease caused by acute inflammatory response has a proven or suspected microbial source [5, 6]. This suspicion is made clinically but the gold standard of its diagnosis has conventionally been blood cultures. The main limitation of blood cultures is the long time usually required for their definite results [7]. Alternative laboratory biomarkers in sepsis have been created since researchers decided to help in its early diagnosis and prognosis. These tests include enzyme-linked immunosorbent assay (ELISA), flucitometry, polymerase chain reaction (PCR), fluorescence in situ hybridization (FISH) and etc. [8-10]. 

Procalcitonin (PCT), is a peptide precursor of calcitonin hormone and a biomarker of bacterial infection [11, 12]. It has a low serum level (0.033 ng/ml) in all healthy individuals, but it would increase up to 1000 times of normal level in acute inflammatory conditions [11, 12]. There are studies approving its high diagnostic accuracy with the sensitivity of 74.8-100% and the specificity of 70-100% [9, 13, 14]. These studies concluded that PCT is a good prognostic marker in sepsis; so it has been used in the evaluation of appropriate treatment response, determination of sepsis severity and estimating the mortality and morbidity rates [12-17]. It has been also proved that D-dimer, as a factor of coagulation, is significantly increased during sepsis [18]. D-dimer has been used as a marker of sepsis severity and most studies revealed that D-dimer is a potent predictor of mortality in sepsis and it is well related to sepsis severity similar to acute physiology and chronic health evaluation II (APACHEII) and simplify acute physiology score II (SAPSII) scores [19-21].

Sepsis-related Organ Failure Assessment score, also known as Sequential Organ Failure Assessment (SOFA) score, is an intensive care unit (ICU) scoring systems. This score is nowadays used to determine whether the patient with sepsis is experiencing organ failure or not [22]. SOFA score can also elucidate the degree of organ failure. The score is based on the involvement of respiratory, cardiovascular, hepatic, coagulation, renal and neurological systems. Each subcategory has 4 scores. The mean and also the highest SOFA score predict sepsis outcomes. It has been shown that an increase in SOFA score in the first 24 to 48 hours estimates a mortality rate of at least 50 to 95%. Scores less than 9 has a predictive mortality rate of 33% while more than 11 has the rate above 95% [23-26]. In this study, we decided to determine the validity of PCT and D-dimer biomarkers in predicting the mortality and severity of sepsis patients referring to the emergency department (ED) based on SOFA score.

## Materials and Methods

*Study design*

This was a cross-sectional study done at Imam Khomeini and Shariati hospitals’ EDs, Tehran, Iran during 2013-2014. The study was approved by the ethics committee of Tehran University of Medical Sciences. The study aim was explained for patients and informed written consent was taken from all of them before participation.

*Study population*

We enrolled patients older than 18 years old with clinical and laboratory findings of sepsis, admitted at the ED. We excluded patients with comorbidities that may interfere with D-dimer measurement including coronary artery disease (CAD), pulmonary thromboemboly (PTE), deep venous thrombosis (DVT) and cancers. 

*Data gathering*

Patients’ demographic data, vital signs, Glasgow coma scale (GCS), arterial blood gas (ABG), serum bilirubin and creatinine level, complete blood count (CBC) were evaluated and recorded in some datasheet designed earlier. PCT concentration was measured by an amplified Cryptate emission technology assay (ROSHE, ELECSYS, 2010, Germany) and its pathologic range for sepsis was more than 2 ng/ml in adults. D-dimer was measured by a Turbidimetric immunoassay in an ACL Elite coagulometer (Barcelona, Spain) using a Hemosil kit (Instrumentation Laboratory, Boston, MA) and values below 500 ng/mL were considered normal. PCT and D-dimer were measured within 1 hours after patients’ admission. Sepsis severity was scored by SOFA score. Patients length of stay and 28-day-mortality rate were registered and followed by phone contact and referring to their medical files.

*Primary and secondary endpoints*

Our primary outcome was to determine the relationship of PCT and D-dimer level in predicting sepsis 28-day-mortality. Our secondary outcomes were comparison of the level of these 2 biomarkers with SOFA score and patients’ length of stay at hospital. 

*Statistical analysis*

The data are presented as frequency (number and percentage), mean [standard deviation (SD)] and median [Interquartile range (IQR)], as appropriate. Variables were tested for normality (Kolmogorov–Smirnov test) before analysis. Analytical statistical tests included the unpaired, two-tailed t-test for continuous normally distributed data and the Mann–Whitney U test for non-normal and ordinal data. The Chi-square and Fisher’s exact tests were used to compare proportions of the qualitative variables. Univariate logistic regression analysis was used as appropriate. Also, we used Pearson or Spearman correlation coinfections for assess correlation between numerical variable (PCT, SOFA, D-dimer and length of hospital stay), based-on normality distribution. The level of significance was 0.05. Statistical package for social sciences (SPSS Inc., Chicago, Illinois, USA) for Windows software (version 22) was used for all data analysis. 

## Results

In this study 87 patients were eligible with clinical suspicion of sepsis in the ED during study period. Finally, we evaluated 64 patients fulfilling our inclusion criteria and 23 patients were excluded: 17 had different types of cancer, 4 had CAD and 2 had PE or DVT. Sixty-four patients with the mean age of 78.3±11.6 were included of whom 30 patients (46.9%) were female and 34 (53.1%) were male. In our study, 28-day-mortality rate was 11 cases (17%). Thus, patients were divided in to 2 groups; the survived group (53 cases) and the expired group (11 cases).

Most study variables did not have a normal distribution. Thus, the median is reported in Table 1. Mann-Whitney U test showed that there is significant difference between two groups based on age (*p*=0.01) and platelet count (*p*=0.02). Logistic regression analysis proved that the effects of age and platelet count were significant on the mortality rate (*p*-value=0.00 and 0.04 respectively). Each year increase in age increased odds mortality rate up to 1.14 times. Each 10000-unit increase in platelet, reduced odds mortality rate up to 0.89 times.

The correlation between PCT and SOFA score was not significant in our study (*p*=0.46). D-dimer also didn’t have any significant correlation with SOFA score (*p*=0.91). We then divided patients into 2 groups, patients with SOFA score less than 10 (with lower mortality rate) and ones with SOFA score more than 10 (with higher mortality rate) based on previous studies [21-23]. We didn’t find any significant association based-on Mann-Whitney U between these groups and PCT or D-dimer (*p*=0.13 and 0.72 respectively). Also, based-on Fisher’s exact test these groups of SOFA score had no statistically significant a with 28-day-mortality rate (*p*=0.51). Based-on Spearman correlation test SOFA score had no correlation with the length of hospital stay (*p*=0.75). Also, the PCT and D-dimer had no correlation with the length of hospital stay (*p*=0.21 and 0.23 respectively).

**Table 1:** Comparison of the study variables between the survived and expired groups

**Variable **		**Survived**	**Expired**	**P**
		**Median (95% CI)**	**Median (95% CI)**	
**Gender**	**Male **	27 (-)	7 (-)	0.33
	**Female**	26 (-)	4 (-)	
**Age (Year old)**		61 (26-84)	80 (68-85)	0.01
**MAP** ^a^ ** (mmHg)**		72 (66-84)	69 (63-82)	0.33
**Vasopressor administration (no)**		7 (-)	9 (-)	0.24
**GCS** ^b^ ** (no/15)**		12 (9-13)	13 (10-13)	0.21
**PaO2/FiO2 (mmHg)**		380 (330-410)	400 (350-415)	0.32
**WBC** ^c^ ** (no/** **µ** **l)**		11230 (1700-22260)	8900 (3390-26900)	0.37
**PLT** ^d^ ** (no/** **µ** **l)**		200000 (6000-606000)	129000 (6100-210000)	0.02
**Hb** ^e^ ** (gr/dl)**		11.2 (4-19.5)	9.3 (4.5-14.3)	0.09
**ESR** ^f^ ** (mm/hr)**		55 (5-150)	40 (10-83)	0.12
**CRP** ^g^ ** (mg/l)**		56 (5-495)	51 (5-115)	0.25
**Crh** ^h^ ** (mg/dl)**		1.4 (0.4-10.3)	1.2 (0.7-3.6)	0.22
**Bili T** ^i^ ** (mg/dl)**		0.8 (0.2-33.7)	0.9 (0.3-5.10)	0.19
**Bili D** ^j^ ** (mg/dl)**		0.2 (0.1-15.7)	0.3 (0.1-2.3)	0.18
**PCT** ^k^ ** (ng/ml)**		0.5 (0.5-4.5)	0.6 (0.3-13.3)	0.22
**D-dimer (ng/ml)**		480 (109-4367)	714 (106-1471)	0.34
**Hospital length of stay (Day)**		6 (1-10)	3 (2-9)	0.18
**SOFA** ^l^ ** score (no/24)**		6 (2-10)	7 (3-9)	0.78

^a^MAP: Mean Arterial Pressure; ^b^GCS: Glasgow Coma Scale; ^c^WBC: White Blood Cell; ^d^PLT: Platelet; ^e^Hb: Hemoglobin; ^f^ESR: Erythrocyte Sedimentation Rate; ^g^CRP: C-reactive Protein; ^h^Cr: Creatinine; ^i^Bili T: Bilirubin Total; ^j^Bili D: Bilirubin Direct; ^k^PCT: Procalcitonin; ^l^SOFA: Sepsis-related Organ Failure Assessment score.

## Discussion

Despite the improvements happened in the diagnosis and treatment of sepsis, it is still one of the main causes of morbidity and mortality in most EDs. The annual incidence of sepsis has a wide range from 300 to 1000 cases/ 100,000 persons [27]. Most of patients with sepsis are hospitalized. The etiology is multifactorial and the incidence is increasing especially among elderlies [28].

In our study sepsis had a mortality rate of 17% which was less that similar studies done in this field (16-56%) [1, 28-35]. We found that patients who had died of sepsis, were significantly older than ones who survived. It is obvious that older patients have more comorbidities than younger patients thus they are more susceptible to mortalities and morbidities caused by sepsis. This study showed that lower platelet count was significantly related to more mortality rate. Yang et al reported that in patients with higher mortality rate, the platelet count was significantly decreased [36]. Mihajlovic *et al*. also announced that the platelet count could independently predict the mortality rate and they showed that this count was lower in patients who had died of sepsis [37]. 

Our main objective was to determine the relationship of PCT and D-dimer level with sepsis mortality rate and severity based on SOFA score. Except age and platelet count, we didn’t find any significant relation between assessed variables and the 28-day mortality rate. As mentioned, PCT and D-dimer had no significant differences between the expired and survived groups. An important point exists here; PCT was checked serially in some studies and its change during follow-ups was evaluated. Podder *et al*. revealed that PCT could change significantly when it was checked serially in sepsis in patients who expired [38]. Zu *et al*. also showed that the baseline PCT had no significant change between the 2 groups [39]. As it is mentioned in Liu *et al*. study, there are many heterogeneities in the time PCT is checked [40].

Yang showed that there were no significant differences in the SOFA or APACHE II scores, CRP, WBC or Cr level between the expired and survived groups along the study. By the contrary, they found that PCT levels at 3^rd^ and 4^th^ weeks were significantly more in the expired group [36]. This study concluded that PCT levels at 3^rd^ and 4^th^ weeks were a good prognostic factor for mortality in sepsis.

Halim et al showed that SOFA score could effectively predict sepsis prognosis and they showed that PCT change has no significant difference between the expired and survived groups [41]. Azevendo *et al*. concluded that 48-hour-SOFA and PCT clearance within the first 24 and 48 hours were good and reliable predictors in sepsis. They revealed that SOFA score was significantly lower in the survived group than the expired group [42]. It was briefly discussed in our paper that the previous studies that just measured the PCT could not find proper and reasonable correlation, but those measured the serum level of PCT serially, reported that PCT level could change significantly in sepsis patients who died. On the other hand, it should be mentioned that, the PCT or D-dimmer level not a cause but may consider a marker in predicting the death. And apparently, their serum level could affect with various known and unknown factors during patients’ hospitalization. So this aspect has lots of no-answer questions yet that need further studies to reply. So the research is still on.

We note some limitations to our study. As it was mentioned, patients with CAD, PTE, DVT and cancers; Sure, these conditions will interfere with D-dimers, but such patients are probably more sick and more likely to die from sepsis. But we need to perform a fundamental study to find the correlation, and if seen, then we can design another study considering the role of underlying disease on the outcome of the patients. The other limitation was that our small sample size might be the reason that we could not find any significant relation between the study variables and outcome. Also, it would be better to check PCT level serially.

In conclusion, the current study failed to demonstrate any significant relation between Procalcitonin or D-dimer with 28-day-mortality rate or severity of sepsis based on SOFA score in critically ill patients with sepsis. Further studies are required to shed light on the issues and complete the results of the current study. 
